# Postnatal Persistent Infection with Classical Swine Fever Virus and Its Immunological Implications

**DOI:** 10.1371/journal.pone.0125692

**Published:** 2015-05-04

**Authors:** Sara Muñoz-González, Nicolas Ruggli, Rosa Rosell, Lester Josué Pérez, Maria Teresa Frías-Leuporeau, Lorenzo Fraile, Maria Montoya, Lorena Cordoba, Mariano Domingo, Felix Ehrensperger, Artur Summerfield, Llilianne Ganges

**Affiliations:** 1 Centre de Recerca en Sanitat Animal (CReSA), IRTA-Universitat Autònoma de Barcelona (UAB), Campus de la UAB, Bellaterra, Barcelona, Spain; 2 Institute of Virology and immunology (IVI), Mittelhäusern, Switzerland; 3 Departament d'Agricultura, Ramaderia, Pesca, Alimentació i Medi Natural, (DAAM), Generalitat de Catalunya, Spain; 4 Centro Nacional de Sanidad Agropecuaria (CENSA), La Habana, Cuba; 5 Departament de Producció Animal, ETSEA, Universidad de Lleida, Lleida, Spain; 6 Institut de Recerca i Tecnologia Agroalimentaria (IRTA), Barcelona, Spain; 7 Departamento de Sanitat i d’Anatomia Animals, Facultat de Veterinària, UAB, Bellaterra-Barcelona, Spain; 8 Institute of Veterinary Pathology, Division of Immunopathology, University Zürich, Zürich, Switzerland; The Ohio State University, UNITED STATES

## Abstract

It is well established that trans-placental transmission of classical swine fever virus (CSFV) during mid-gestation can lead to persistently infected offspring. The aim of the present study was to evaluate the ability of CSFV to induce viral persistence upon early postnatal infection. Two litters of 10 piglets each were infected intranasally on the day of birth with low and moderate virulence CSFV isolates, respectively. During six weeks after postnatal infection, most of the piglets remained clinically healthy, despite persistent high virus titres in the serum. Importantly, these animals were unable to mount any detectable humoral and cellular immune response. At necropsy, the most prominent gross pathological lesion was a severe thymus atrophy. Four weeks after infection, PBMCs from the persistently infected seronegative piglets were unresponsive to both, specific CSFV and non-specific PHA stimulation in terms of IFN-γ-producing cells. These results suggested the development of a state of immunosuppression in these postnatally persistently infected pigs. However, IL-10 was undetectable in the sera of the persistently infected animals. Interestingly, CSFV-stimulated PBMCs from the persistently infected piglets produced IL-10. Nevertheless, despite the addition of the anti-IL-10 antibody in the PBMC culture from persistently infected piglets, the response of the IFN-γ producing cells was not restored. Therefore, other factors than IL-10 may be involved in the general suppression of the T-cell responses upon CSFV and mitogen activation. Interestingly, bone marrow immature granulocytes were increased and targeted by the virus in persistently infected piglets. Taken together, we provided the first data demonstrating the feasibility of CSFV in generating a postnatal persistent disease, which has not been shown for other members of the Pestivirus genus yet. Since serological methods are routinely used in CSFV surveillance, persistently infected pigs might go unnoticed. In addition to the epidemiological and economic significance of persistent CSFV infection, this model could be useful for understanding the mechanisms of viral persistence.

## Introduction

Classical swine fever (CSF) is a highly contagious viral disease of domestic pigs and wild boars [1], which has caused major losses in stock farming [2]. The causative agent, CSF virus (CSFV), is a member of the genus *Pestivirus* within the family *Flaviviridae* [1]. CSFV is composed of a lipid envelope, a capsid and a single plus-strand RNA genome carrying a single, large open reading frame (ORF) flanked by two untranslated regions (UTRs). The ORF encodes a polyprotein of approximately 3900 amino acids, which are processed by cellular and viral proteases in the four structural proteins C, E^rns^, E1, E2 and in the 8 non-structural proteins N^pro^, P7, NS2, NS3, NS4A, NS4B, NS5A, and NS5B [3].

Although CSF has been widely eradicated, it remains endemic in certain areas of Asia, Europe, Central and South America, and parts of Africa [4–10], representing a constant threat to the pig industry. Depending on the virulence of the strain, varying degrees of disease severity have been observed, ranging from acute or chronic to subclinical forms [7,11,12]. In general, while infections with virulent strains result in acute haemorrhagic disease, the infection caused by less virulent isolates can become chronic or subclinical [11,13]. Pigs infected with low virulent strains can shed the virus continuously or intermittently for months, representing a constant source of reinfection in endemic areas and a threat to virus-free countries [4,14]. Interestingly, in endemic areas, such as Cuba and China, a trend towards milder, chronic clinical manifestations of CSF has been observed [4,5,15]. It was suggested that CSFV evolution towards low virulent viruses in these regions was driven in part by a positive selection pressure linked to inefficient vaccination programs, leading to mostly unapparent clinical manifestations. Therefore, these viral strains are of great significance in endemic countries [4,5,16,17]. However, the pathogenesis and disease progression after infection with low virulent CSFV isolates are poorly understood.

The occurrence of low virulence CSFV strains in the field and their role in the “pregnant carrier sow syndrome” and in congenital infection of the foetus by trans-placental transmission have been extensively described [18–21]. There has been, however, some controversy over the importance of such congenital persistent infections in virus dissemination [22,23]. Numerous reports on experimental congenital infections have shown that congenitally persistently infected piglets result mostly from infection during mid-gestation [13,20,21,24,25]. However, the pathogenesis of this persistence is not completely understood and has been related to a specific immunotolerance to CSFV [19,25–27]. At birth, congenitally persistently infected piglets are often not recognised as infected animals, appearing healthy and developing a runting-like syndrome only later, with lesions that are not characteristic of CSF. As opposed to congenital infections, however, there have been few reports only suggesting a possible occasional occurrence of virus persistence after postnatal infection of newborns [28] and after infection of 6-week-old weaned pigs [27,29].

Considering the above premises, the aim of this work was to evaluate the ability of two CSFV field isolates of low and moderate virulence, respectively, of different origins and genotypes to induce viral persistence after early postnatal infection, as well as to study the characteristics of the immunological response related to viral persistence.

## Materials and Methods

### Cells and viruses

PK-15 cells (ATCC CCL 33) were cultured in DMEM medium, supplemented with 10% foetal bovine serum (FBS) pestivirus-free at 37°C in 5% CO_2_. The cells were infected with 0.1 TCID_50_/cell in 2% FBS, and the virus was harvested 48 h later. Peroxidase-linked assay (PLA) [30] was used for viral titration following the statistical methods described by Reed and Muench [31]. The Catalonia 01 (Cat01) strain used in this study was isolated from the CSF Spanish epizootic in 2000–2001 [32]. This isolate belongs to the CSFV 2.3 genogroup [4]. The course of the infection by this strain was found to be mild [32,33]. The Pinar del Rio (PR) strain is a prototype low virulence CSFV isolate circulating currently in Cuba [4]. It was isolated after more than 18.5 years of endemic CSF in Cuba, during which CSFV evolved under constant immunological pressure exerted by suboptimal vaccination [4,34]. Finally, the Thiverval vaccine strain (provided by Pasteur Institute, Romania) was used as stimulus in the Elispot assays for CSFV-specific IFN-γ-producing cells detection. This strain belongs to the CSFV 1.1 genogroup [35].

### Experimental design

Two pregnant, pestivirus-free sows (landrace) of 108 days into gestation were housed in the BSL3 animal facility at CReSA (Barcelona, Spain). Each sow was housed in a separate box with standard facilities to allocate pregnant and lactating sows. Their deliveries were synchronised with d-cloprostenol 75 pg/sow at 114 days of gestation. After 24 hours, the deliveries were initiated, and 10 piglets were inoculated intranasally during the first 8 hours after birth with 2.5 mL of 2.5 x 10^4^ TCID of the PR strain or Cat01 strain. The inoculation of the piglets was conducted separately from their mothers. Additionally, 2 piglets at 6 weeks of age, from a sow of the same origin, served as control, non-inoculated pigs (numbered 32 and 33). The piglets were kept with their mothers during the 6 weeks of the experiment, and they received feed (StartRite, Cargill, Spain) from week 5 onwards. After infection, serum samples were collected every week over the 6 weeks post-infection, and nasal and rectal swabs were obtained at 2, 3, 4, 5 and 6 weeks post-infection. Blood samples for the isolation of PBMCs were obtained at 4 and 6 weeks post-infection, and tissues from the tonsils and thymus were obtained after euthanasia. The procedure for the euthanasia of the animals was based on an accepted method included in European Directive 2010/63/EU, using an anaesthetic overdose of 60–100 mg of pentobarbital per kilogram of weight, administered via the vena cava.

A trained veterinarian recorded the clinical signs daily in a blinded manner. To reduce handling to the litters during the first three days of life, the rectal temperature was recorded from 3 days after infection (after birth) until the end of the trial. The experiments were approved by the Ethics Committee for Animal Experiments of the Autonomous University of Barcelona (UAB) under number 5796, according to existing national and European regulations.

### Detection of CSFV RNA

RNA was extracted from all the samples using the NucleoSpin RNA isolation kit (Macherey-Nagel), according to the manufacturer's instructions. In all cases, RNA was extracted from an initial sample volume of 150 μL to obtain a final volume of 50 μL of RNA, which was stored at -80°C. The presence of CSFV RNA in the serum and in nasal and rectal swabs, as well as tonsil, thymus and bone marrow samples, was analysed by real time (RT)-PCR [36]. This test was used in our laboratory for inter-laboratory comparisons of CSFV diagnoses, organised by the EU Reference Laboratory. Positive results were considered for threshold cycle values (CT) equal to or less than 42. Samples in which fluorescence was undetectable were considered negative.

### Detection of E2-specific and neutralising antibodies

The serum samples were tested with neutralisation peroxidase-linked assay (NPLA) [37], and the titres were expressed as the reciprocal dilution of serum that neutralised 100 TCID_50_ of the Cat01 or PR strain in 50% of the culture replicates. The detection of E2-specific antibodies was performed using a commercial ELISA kit (IDEXX); the samples were considered positive when the blocking percentage was ≥40%, following the manufacturer's recommendations.

### ELISA for IFN-α detection in serum samples

Anti-IFN-α monoclonal antibodies (K9 and K17) and IFN-α recombinant protein (PBL Biomedical Laboratories, Piscataway, New Jersey, USA) were used in an ELISA assay to detect IFN-α in serum samples [38–41]. The cut-off value was calculated as the average of the optical density of negative controls (blank and negative serums before CSFV infection) plus three standard deviations. Cytokine concentrations in serum were determined using a regression line built with the optical densities of the cytokine standards used in the test.

### PBMCs and ELISPOT assay for CSFV-specific IFN-γ-producing cells

Peripheral blood mononuclear cells (PBMCs) were obtained from whole blood collected at 4 and 6 weeks post-infection, and these cells were separated by density-gradient centrifugation with Histopaque 1077 (Sigma). The number and viability of the PBMCs were determined by staining with Trypan Blue [42]. ELISPOT assay to detect CSFV-specific IFN-γ cells was performed as previously described [41]. Briefly, plates (Costar 3590, Corning) were coated overnight with 5 μg/ml of capture antibody (P2G10, Pharmigen). Detection was performed using a biotinylated antibody (P2C11, Pharmigin). A total of 5x10^5^ PBMCs/well were plated in triplicate at 0.1 multiplicity of infection (MOI) of the Cat01 and PR CSFV strains. Moreover, the same samples were incubated in the presence of Thiverval strain at 0.01 MOI and phytohaemagglutinin (PHA) (10 μg/ml). The controls were incubated in the presence of mock-stimulated wells. The numbers of spots in the media for mock-stimulated wells were considered to be the baseline for the calculation of antigen-specific frequencies of IFN-γ-producing cells.

### BMHC collection and phenotype analysis

Bone marrow haematopoietic cells (BMHCs) were obtained from the femurs of selected pigs (pigs 1 and 3: infected with the PR strain; pigs 19 and 23: infected with the Cat01 strain; and two non-infected pigs) at 6 weeks of age, following the protocol previously described [43,44]. To phenotype these cells, flow cytometry was performed using the corresponding hybridoma supernatants in the indirect labelling for SLA-I (74-11-10, IgG2b), SLA-II (1F12, IgG2b), CD163 (2A10/11, IgG1), CD172a (BA1C11, IgG1), granulocyte precursors (6D10, IgG2a), and c-kit or CD117 (2B8/BM IgG1); all of the hybridoma supernatants were kindly donated by Dr. J. Dominguez (INIA, Madrid, Spain). For the detection of CSFV-infected cells, a polyclonal FITC-labelled anti-CSFV conjugate (PrioCON FITC conjugate PAb-CSF, Prionics, Switzerland) was used. For the cellular markers, the secondary antibody was R-phycoerythrin goat anti-mouse IgG (Jackson ImmunoResearch, Suffolk, UK). Briefly, 2.5 × 10^5^ cells/50 μl/well were labelled for 1 h at 4°C. The anti-CSFV conjugate was diluted 1:100 in cold PBS with 2% FBS. After 1 h of incubation at 4°C, the cells were washed with cold PBS with 2% FBS by centrifugation at 450 × *g*, at 4°C for 5 min. Then, the secondary antibody conjugated with R-phycoerythrin diluted 1:200 was added for SLA-I, SLA-II, CD163, CD172a, 6D10 and CD117 markers. The cells were incubated for a further 45 min at 4°C and then were washed as before and resuspended in PBS with 2% FBS. SLA-I, SLA-II, CD163, CD172a, 6D10, CD117, and CSFV-positive cells and unstained cells were counted using FACSaria I (Becton Dickinson), and the data were analysed by FACSDiva software, version 6.1.2. Irrelevant isotype-matched mAbs, unlabelled or labelled with the different fluorochromes, were used as negative controls. The gate strategy was applied in 90% of living cells using the forward and side scatter (FS/SS) characteristics. For two colour immunolabelling, the same procedure described above for incubation and washing was followed. To 2.5 × 10^5^ cells/50 μl/well, 50 μl of SLA-II marker was added, followed by the secondary antibody conjugated with R-phycoerythrin diluted 1:200. Mab CD172a was biotinylated using standard protocols (CD172a_b). After the third wash, the cells were incubated with CD172a_b for 1 h at 4°C. Finally streptavidin-allophycocyanin (APC) was added at a 1:100 dilution. For 6D10^+^/CSFV^+^ labelling, the polyclonal FITC-labelled anti-CSFV conjugate and 6D10 hybridoma supernatants revealed with R-phycoerythrin conjugate were used. CD172a^+^/SLA-II^+^ or 6D10^+^/CSFV^+^ was acquired using FACSAria I (Becton Dickinson, San Jose, California, USA), and the positive percentages were analysed by FACSDiva software, version 6.1.2.

### Sorting of 6D10^+^ cells

The 6D10^+^ cell subsets were sorted using a live sterile cell sorting system (FACSAria, Beckton Dickinson, San Jose, California, USA). To obtain 6D10^+^ cells, 24 x10^6^ BMHCs were incubated with 6D10 hybridoma supernatant for 1 h on ice, washed with PBS containing 2% FBS, and incubated with R-phycoerythrin conjugate goat (Fab′)_2_ anti-mouse Ig (Dako, Denmark). Single cell sorting was performed in using purity precision mode, with a 70 μm nozzle. The fluorescence reading was performed upon excitation with a 488 nm argon laser. The 6D10^+^ and 6D10^−^ cells were more than 95% pure by flow cytometry, and a total of 6701444 6D-10^+^ cells were recovered with 97% efficiency. The presence of CSFV RNA in both types of recovered cells was analysed by RT-PCR [36].

### Stimulated IL-10 production by PBMCs from CSFV-infected pigs

To elucidate the role of IL-10 in postnatal persistence, the levels of IL-10 were firstly determined in the sera from the piglets and sows. These samples were analysed by ELISA (IL-10 Swine ELISA Kit, Life-Technologies, USA) at 7, 14, 21 and 42 days post infection (dpi). In contrast, to measure IL-10 production, 2.5 x 10^6^ PBMCs/mL were cultivated for duplication in the presence or absence of neutralising IL-10 clone (148801, R&D System, USA) at 6 μg/10^6^ cells in RPMI medium with 10% FBS. A total of 2.5x10^5^ PBMCs/well were stimulated at 37°C in 96-well plates with mock, CSFV (Catalonia strain) at 0.1 MOI or PHA (10 μg/ mL). The supernatants were removed after 96 h, and the concentrations of IL-10 were determined by ELISA.

Moreover, to investigate the effect of IL-10 on IFN-γ production, PBMCs collected at 6 weeks p.i. from pigs 23, 25, 27 (CSFV persistently infected pigs), 32 and 33 (non-infected pigs) were cultivated in one ELISPOT assay in the presence or absence of neutralising IL-10 clone (148801, R&D System, USA) at 6 μg/10^6^ cells. These cells were cultivated for duplication and were stimulated with mock, CSFV (Catalonia strain) at 0.1 MOI and PHA (10 μg/mL). These experiments were repeated twice under the same conditions.

### Statistical analysis

All of the statistical analyses were performed using SPSS software, version 15.0 (SPSS Inc., Chicago, Illinois, USA), using "piglet or sow" as the experimental unit. The significance level (α) was set at *P*<0.05. Throughout the trial, a non-parametric test (Mann-Whitney) was chosen to compare values obtained from the immunological parameters between groups (Cat01 or PR strain). This non-parametric analysis was chosen due to the small number of animals used in each experimental group. Finally, Fisher’s exact test was used to test the associations of the percentage of CSFV-positive piglets in serum, nasal and rectal swabs, and the tonsils and thymus among the experimental groups throughout the trial.

## Results

### Clinical signs developed in the newborn infected piglets

**Fig 1:**
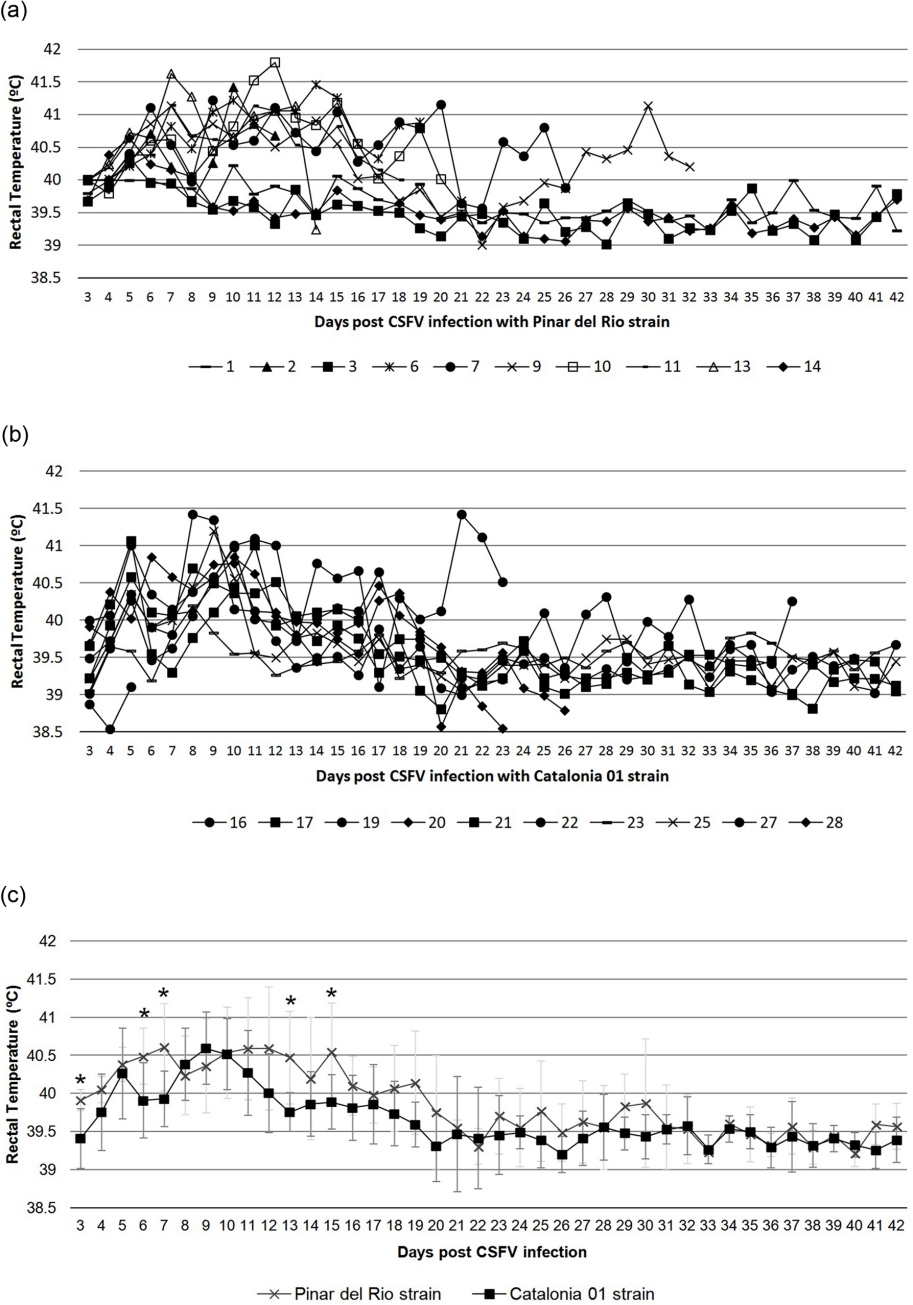
Rectal temperature values (°C) at 3 days post-infection with PR or Cat01 CSFV strains daily during the trial. (a) Individual rectal temperature values (°C) in pigs inoculated with the PR strain (pigs 1 to 14). (b) Individual rectal temperature values (°C) in pigs inoculated with the Cat01 strain (pig 16 to 28). (d) Mean and standard deviation values of the rectal temperature (°C) per group (PR and Cat01) per day. Cross symbol shows the mean value for PR group. The square symbol shows the mean value for Cat01 group. The light grey bars indicate the standard deviation value for PR group. Dark grey bars indicate the standard deviation value for Cat01 group. Values greater than 40°C were considered to indicate fever. An asterisk indicates a statistically significantly higher rectal temperatures in piglets infected with the PR strain, compared with the levels found in piglets infected with the Cat01 strain (*P*<0.05).

**Fig 2:**
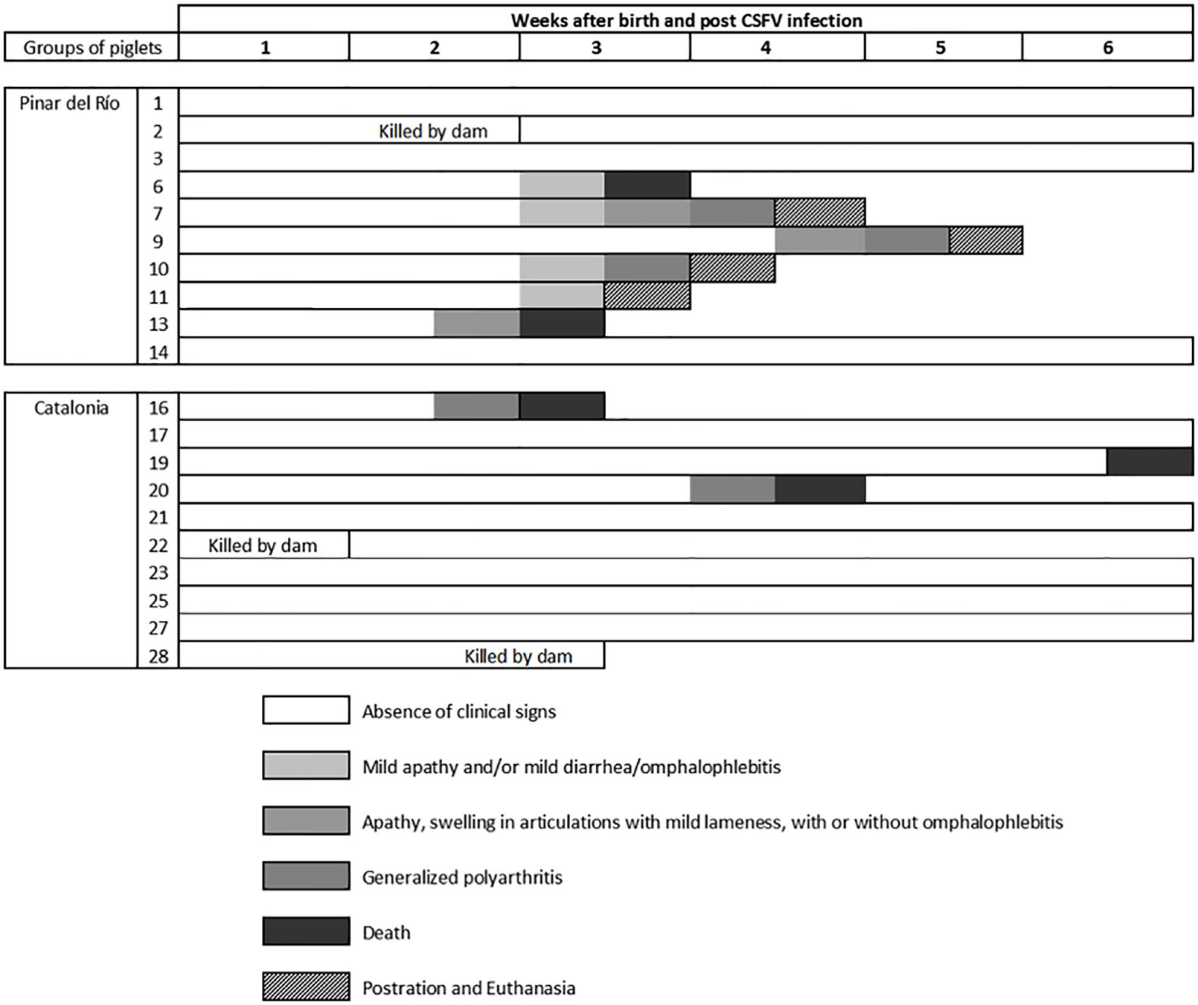
Individual clinical signs in piglets after early postnatal infections with CSFV PR or Cat01 field isolates. The piglets were monitored daily over the 6 weeks of the study. The evolution in the development of clinical signs is represented by an intensity colour scale (from low to high).

The pathogenicity of the two CSFV field isolates was studied in newborn piglets. To this end, two litters of 10 piglets each were inoculated intranasally on the day of their birth with 2.5 x 10^4^ TCID_50_ of CSFV strains PR and Cat01, respectively. The infected piglets were kept with the dam for 6 weeks and were monitored for clinical symptoms, virus replication and immune responses against CSFV. In the PR and Cat01 group, three and five animals, respectively, did not show visible clinical signs over the 6 week duration of the experiment. Fever peaks during the first 15 days post-infection were detected in the infected animals, especially in PR infected group. In addition, similar profiles in the rectal temperature values were found for both groups during the trial. Only 5 days (3, 6, 7, 13 and 15 dpi) were significantly different between the groups (Figs 1 and 2).

Six of PR and three of Cat01 animals died or were euthanised between the 3^rd^ and 6^th^ weeks after CSFV inoculation after they suddenly become apathic and developed severe secondary infections, resulting in omphalophlebitis, mild diarrhoea, polyarthritis or prostration (Fig 2). Finally, one apparent healthy piglet from the PR group and two from the Cat01 group were lost during the second, first and third weeks, respectively, killed by their dams (Figs 1 and 2). Interestingly, irrespective of the outcome of CSFV infection, the thymus was very small at the time of necropsy in all of the piglets except for pigs 1, 3 and 14 from the PR group (data not shown).

### The majority of the newborn CSFV-inoculated piglets did not clear the virus

**Fig 3:**
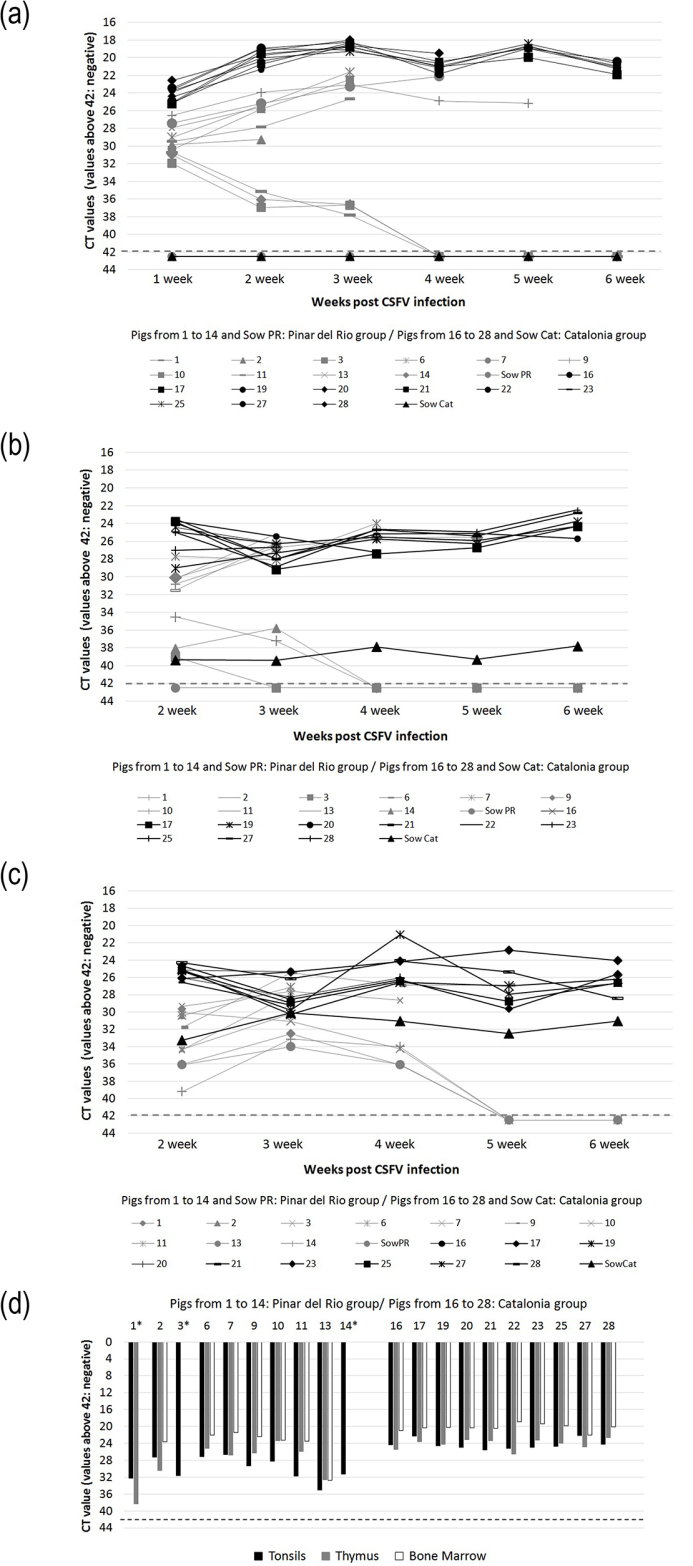
Detection of CSFV RNA through real-time PCR in serum and nasal and rectal swabs. Detection of CSFV RNA through real-time PCR in serum (a) and nasal (b) and rectal (c) swabs. The piglets and the sows infected with the PR and the Cat01 strains are represented in grey and black colours, respectively. Positive results were considered for CT values equal to or less than 42. (d) Detection of CSFV RNA through RT-PCR in the tonsils, thymus and bone marrow are represented in black, grey and white colours, respectively. Positive results were considered for CT values equal or less than 42. An asterisk indicates negative results in pigs 1, 3 and 14 in some of the tissues analysed.

CSFV replication after perinatal inoculation of the piglets with the PR and Cat01 strains was monitored by qRT-PCR for viral RNA at weekly intervals in serum, nasal and rectal swabs and in the tonsils, thymus and bone marrow at necropsy (Fig 3). Despite the absence of clinical signs, all of the piglets had strong viral RNA signals in their serum 7 days after inoculation. At later time points, the viral RNA load further increased and remained high until the end of the experiment or until death or euthanasia in all of the piglets infected with Cat01 and in 7 of 10 piglets infected with PR. The three remaining piglets in the PR group (pigs 1, 3 and 14) cleared the virus from their circulation by day 28 post-inoculation, as determined by real time RT-PCR (Fig 3A). Interestingly, the Cat01 virus-infected piglets had significantly higher CSFV RNA levels in their serum than the PR virus-infected piglets (*P* = 0.0002), which might indicate a lower virulence of the PR strain. Virus titration at 21 and 28 days after inoculation confirmed the qRT-PCR data, with overall higher virus titres in the serum of the Cat01-infected *versus* the PR-infected piglets (*P* = 0.006, Table 1). Additionally, the three PR-infected piglets that had undetectable viral RNA at day 28 post-inoculation were also negative for virus isolation already at day 21 (Table 1 and Fig 3). However, in the two sows, the virus could not be detected in the serum, either by qRT-PCR or by virus isolation at any time during the experiment, confirming the low virulence of the two strains. Nevertheless, the dam of the Cat01-infected piglets had low levels of viral RNA detectable in the nasal and rectal swabs until the end of the experiment, while the PR-infected sow remained negative from the swabs (Fig 3B and 3C). CSFV RNA was detected in the nasal and rectal swabs of all of the inoculated piglets until the end of the experiment or the day on which they were euthanised, except for the 3 PR-infected piglets that recovered from infection and stopped shedding the virus by day 28 (Fig 3B and 3C). The viral RNA load in the nasal and rectal swabs from the pigs infected with the Cat01 strain was higher than for the PR-infected piglets at 14 days p.i. only. At necropsy, CSFV RNA was detected in the tonsils, thymus and bone marrow of all of the piglets infected with Cat01 strain and from the 7 PR virus-infected piglets that did not clear the virus (Fig 3D). It is worth noting that the CSFV RNA levels detected in the tonsils, thymus and bone marrow were significantly higher in the Cat01 group than in the PR group (tonsils, *P* = 0.0002; thymus, *P* = 0.009; bone marrow, *P* = 0.001). Interestingly, the two sows were also qRT-PCR-positive in the tonsils (data not shown). Altogether, 85% of the piglets inoculated on the day of their birth were not capable of clearing the virus within the 4 to 6 weeks of their lifespans.

**Table 1:** Virus isolation and virus titration in PK-15 cells with serums samples at 3 and 4 weeks post-infection.

		3 week post-infection		4 week post-infection	
Inoculum	Pig number	Virus isolation	Virus titration^a^	Virus isolation	Virus titration^a^
Pinar del Rio strain	1	Negative	Negative	Negative	Negative
	3	Negative	Negative	Negative	Negative
	6	+	10 ^5. 5^	†	
	7	+	10 ^6. 35^	**+**	10 ^7.00^
	9	+	10 ^5.66^	**+**	10 ^5. 57^
	10	+	10 ^6. 35^	†	
	14	Negative	Negative	Negative	Negative
	Sow PR	Negative	Negative	Negative	Negative
Catalonia strain	16	**+**	10 ^6.66^	†	
	17	**+**	10 ^6. 57^	**+**	10 ^6. 20^
	19	**+**	10 ^7.12^	**+**	10 ^6.80^
	20	**+**	10 ^6.66^	**+**	10 ^7.16^
	21	**+**	10 ^6. 57^	**+**	10 ^6.49^
	23	**+**	10 ^6.66^	**+**	10 ^6.66^
	25	**+**	10 ^6.75^	**+**	10 ^6.71^
	27	**+**	10 ^6. 57^	**+**	10 ^7.00^
	28^b^	**+**	10 ^7.00^	†	
	Sow Cat	Negative	Negative	Negative	Negative

Negative: The virus isolation was negative.

Symbol +: The virus isolation was positive.

Symbol †: Dead

^a^: Virus Titration in TCID 50/mL

^b^: sample from pig 28 analysed at 19 dpi

### The newborn piglets incapable of clearing CSFV did not seroconvert

**Fig 4:**
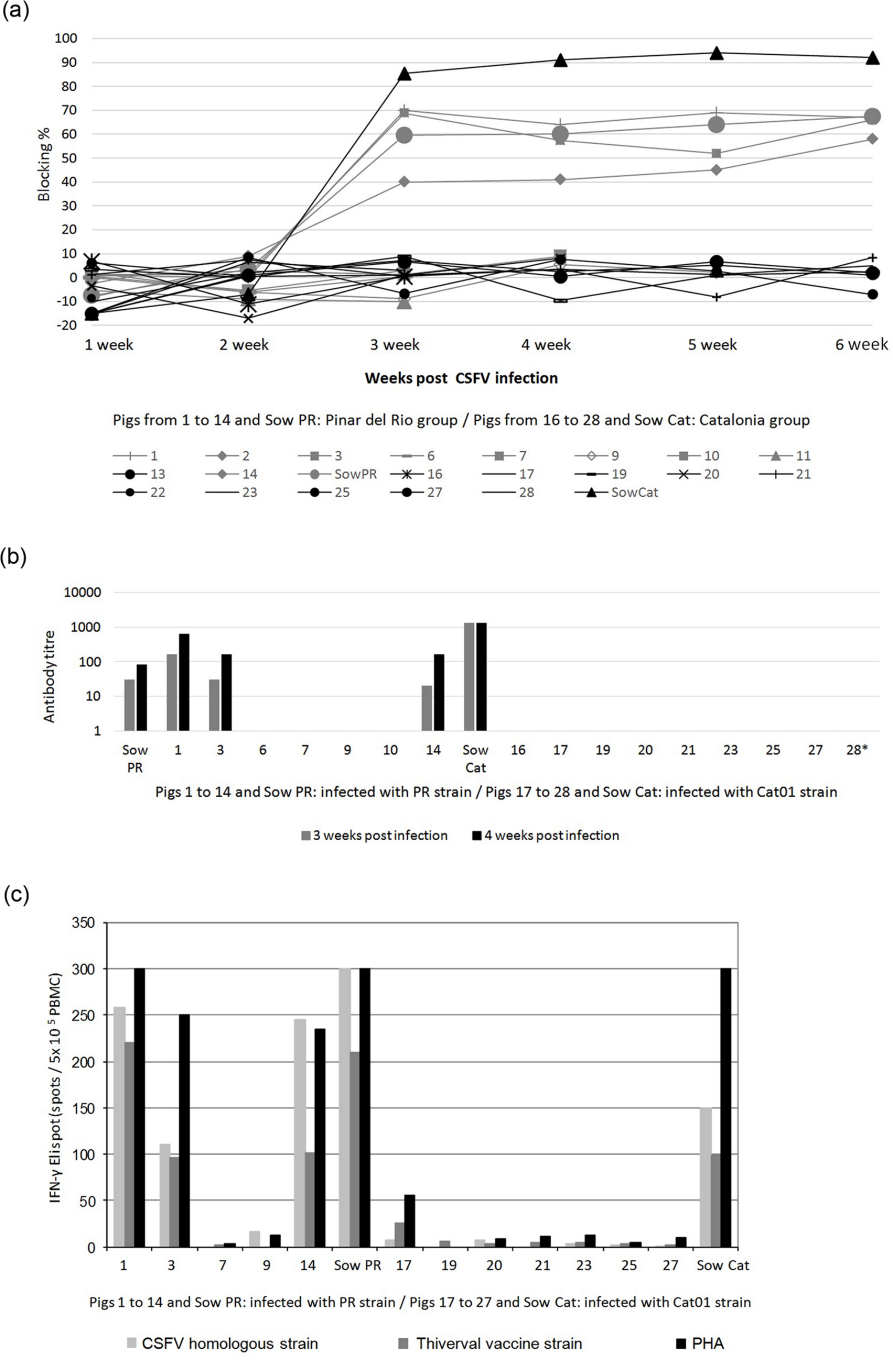
Humoral and cellular immune response against CSFV infection. (a) Antibody response to E2 glycoprotein detected by ELISA (IDEXX) after infection (in blocking %). Values greater than 40% blocking were considered positive. (b) Neutralising antibody titres against PR (pigs 1 to 14 and sow PR) and Cat01 (pigs 16 to 28 and sow Cat) CSFV strains at 3 and 4 weeks p.i. (c) Lack of IFN- γ response by ELISPOT assay in CSFV postnatally persistently infected piglets and detection of effective response in immunocompetent pigs from the PR group at 4 weeks p.i. A total of 5x10^5^ PBMCs/well were plated in triplicates at 0.1 multiplicity of infection (MOI) of CSFV homologous strains: PR strain (samples from pigs 1 to 14 and sow PR) and Cat01 strain (samples from pigs 17 to 27 and sow Cat). Moreover, the samples were incubated in the presence of Thiverval strain at 0.01 MOI and phytohaemagglutinin (PHA) (10 μg/ml). Asterisk symbol indicates the sample from pig 28 analysed at 19 dpi.

To determine whether the inability to clear CSFV was related to a deficient humoral immune response, serum samples from the infected and control pigs were analysed weekly for CSFV-specific antibodies. No detectable antibody response were found in the control pigs (data not shown). Likewise, there were no detectable antibody responses after infection in any of the piglets infected with the Cat01 strain or in the 7 piglets infected with the PR strain that did not clear the virus during the six weeks of the experiment or until death or euthanasia (Fig 4A and 4B). In contrast, the 3 piglets (#1, 3 and 14) that cleared the PR virus were positive for E2-specific antibodies and virus-neutralising antibodies from 21 days p.i. onwards. The sows were also positive for both binding and neutralising antibodies (Fig 4A and 4B). Interestingly, the Cat01 virus induced a stronger antibody response than the PR virus in the sows, which again might be related to the higher replication rate of the Cat01 virus.

### CSFV-specific IFN-γ-producing cells were lacking in the piglets incapable of clearing CSFV

PBMCs from all of the piglets and from the two sows were analysed for virus-specific and-non-specific IFN-γ responses by ELISPOT assay at four weeks p.i. Very few IFN-γ-producing cells were found upon CSFV and PHA stimulation of PBMCs from all 7 of the surviving piglets in the Cat01-infected group and from the two surviving piglets from the PR-infected group that had not cleared the virus (Fig 4C). In contrast, a large proportion of CSFV-specific and PHA-responsive IFN-γ-producing cells were detected in the PBMCs from the three PR-infected piglets that had seroconverted and from the two sows (Fig 4C). In conclusion, these data altogether showed that early postnatal infection of piglets with low virulence CSFV could result in virus persistence due to a lack of B- and T-cell responses. The lack of responsiveness of PBMCs to PHA suggested that the persistently infected piglets were immunosuppressed.

### All of the piglets responded to CSFV infection with IFN-α production

**Fig 5:**
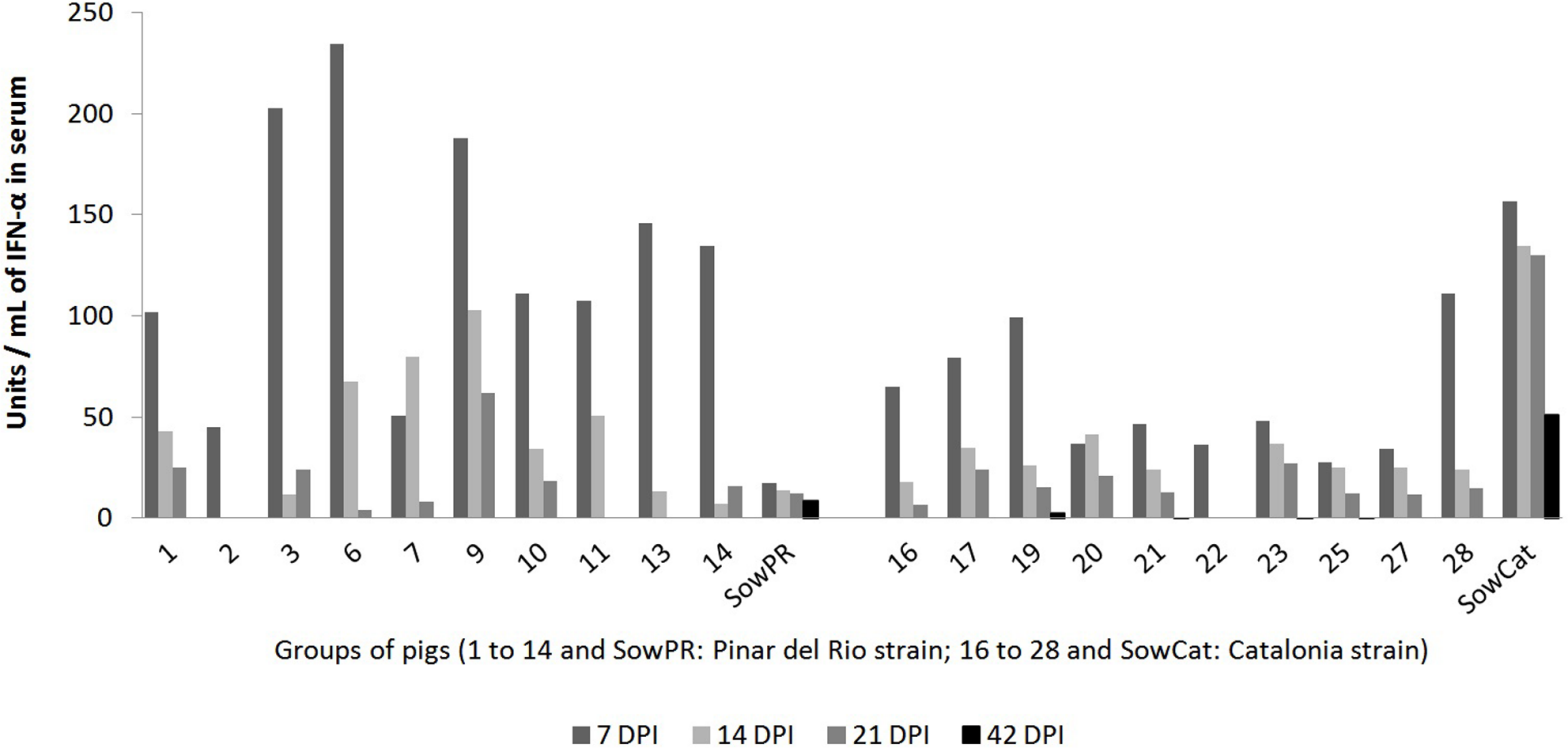
Serum IFN-α levels in the piglets infected with the CSFV strains PR or Cat01. Serum IFN-α levels in the piglets infected with the CSFV strains PR or Cat01 at four different times p.i. (7, 14, 21 and 42 days post-infection (DPI)). IFN-α levels were statistically significantly higher in piglets infected with the PR strain, compared with the levels in the piglets infected with the Cat01 strain (*P*<0.05).

After having shown that the adaptive immune response of the persistently infected piglets was clearly impaired, we wondered whether their innate immune responses were also affected. Thus, serum IFN-α levels in the persistent and immunocompetent piglets and the sows were analysed by ELISA at 7, 14, 21 and 42 dpi. Considerable but variable levels of serum IFN-α were found in all of the animals mainly at day 7 p.i. and in some of the PR virus persistently infected piglets at 14 and 21 d.p.i. (Fig 5). The mean IFN-α content was significantly higher in the piglets infected with the PR strain than in the piglets infected with the Cat01 strain (*P* = 0.0046), although viraemia was lower. Interestingly, the three immunocompetent piglets responded with IFN-α similar to that of the persistently infected animals. Interestingly, the two sows exhibited different IFN-α patterns than the piglets, with the Cat01-infected sow having a higher concentration of IFN-α in the serum than the sow infected with the PR strain (Fig 5). These results showed that the IFN-α response was not affected in the persistently infected piglets, as opposed to the adaptive humoral and cellular immune responses.

### The bone marrow 6D10^+^ immature granulocytes were increased and targeted by CSFV in persistently infected piglets

**Fig 6:**
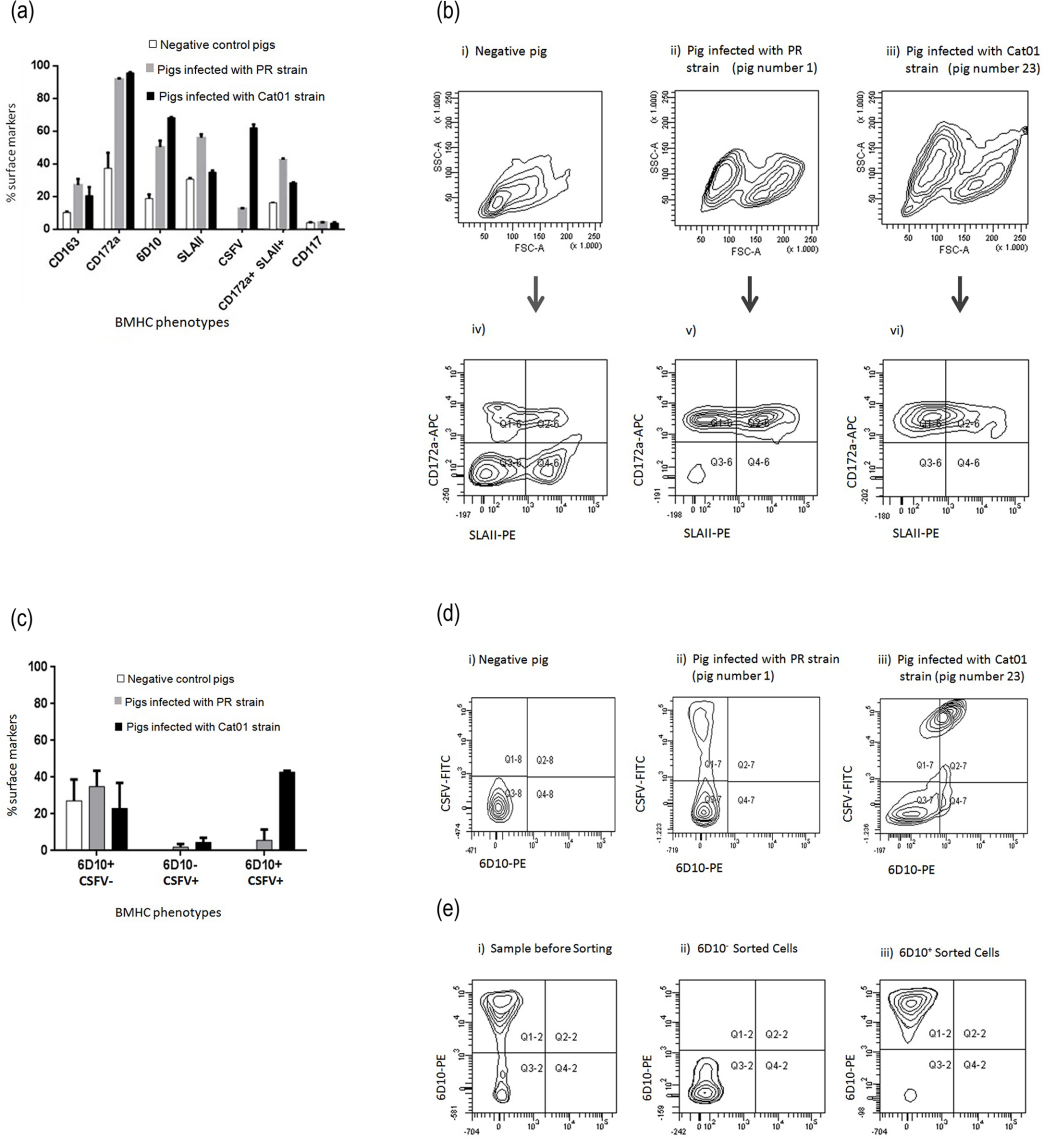
Expression of cellular markers and comparative phenotypes of BMHCs from PR, Cat01 and non-infected pigs. (a) Expression of 5 different surface markers and CSFV in BMHCs from infected and non-infected pigs. (b) Comparative phenotypes of BMHCs obtained from non-infected pigs (i, iv) and pigs infected with the PR (ii, v) and Cat01 (iii, vi) strains. (i, ii and iii): forward scatter (relative cell size, x-axis) and side scatter (relative granularity, y-axis). (iv, v and vi): double labelling immunofluorescence image of the BMHCs from (i, ii and iii), in terms of CD172a common myeloid marker (y-axis) and SLA-II (x-axis). (c) Percentage of granulocyte 6D10^+^ and 6D10^+^_CSFV^+^ double-positive cells in the BMHCs. (d) Double labelling immunofluorescence image of the BMHCs from one non-infected and two infected pigs, in terms of 6D10, immature granulocytes marker (x-axis) and CSFV (y-axis). (e) Sorting of 6D10^+^ BMHCs. These experiments were repeated twice under the same conditions.

The data suggested that immunosuppression occurred at a certain point in the adaptive immune response. Therefore, we wondered whether myeloid bone marrow cells were affected by viral persistence. The fate and infection of the myeloid BMHCs were determined at necropsy in 6-week-old (i) non-infected piglets, (ii) seropositive piglets infected with the PR virus (pigs 1 and 3) and (iii) seronegative piglets infected with the Cat01 virus (pigs 19 and 23). The percentages of myeloid (CD172a^+^, CD163^+^) cells were clearly increased in both the PR and Cat01 virus-infected piglets (Fig 6). In contrast, the increases in SLAII^+^ and CD172a^+^/SLAII^+^ cells were more prominent in the immunocompetent piglets infected with the PR strain. Consistent with viral persistence, the percentage of CSFV-positive BMHCs overall was three times higher in the pigs infected with Cat01, compared with the PR virus-infected piglets that had seroconverted to the infection (Fig 6A and 6B). Accordingly, a higher percentage of immature granulocytes (6D10^+^) and CSFV^+^/6D10^+^ cells was found in the BMHCs from the Cat01 virus-infected piglets (Fig 6C and 6D). This finding was confirmed by qRT-PCR with 6D10^+^ sorted cells (Fig 6E).

### CSFV induced high IL-10 production in PBMCs from persistently infected piglets

**Fig 7:**
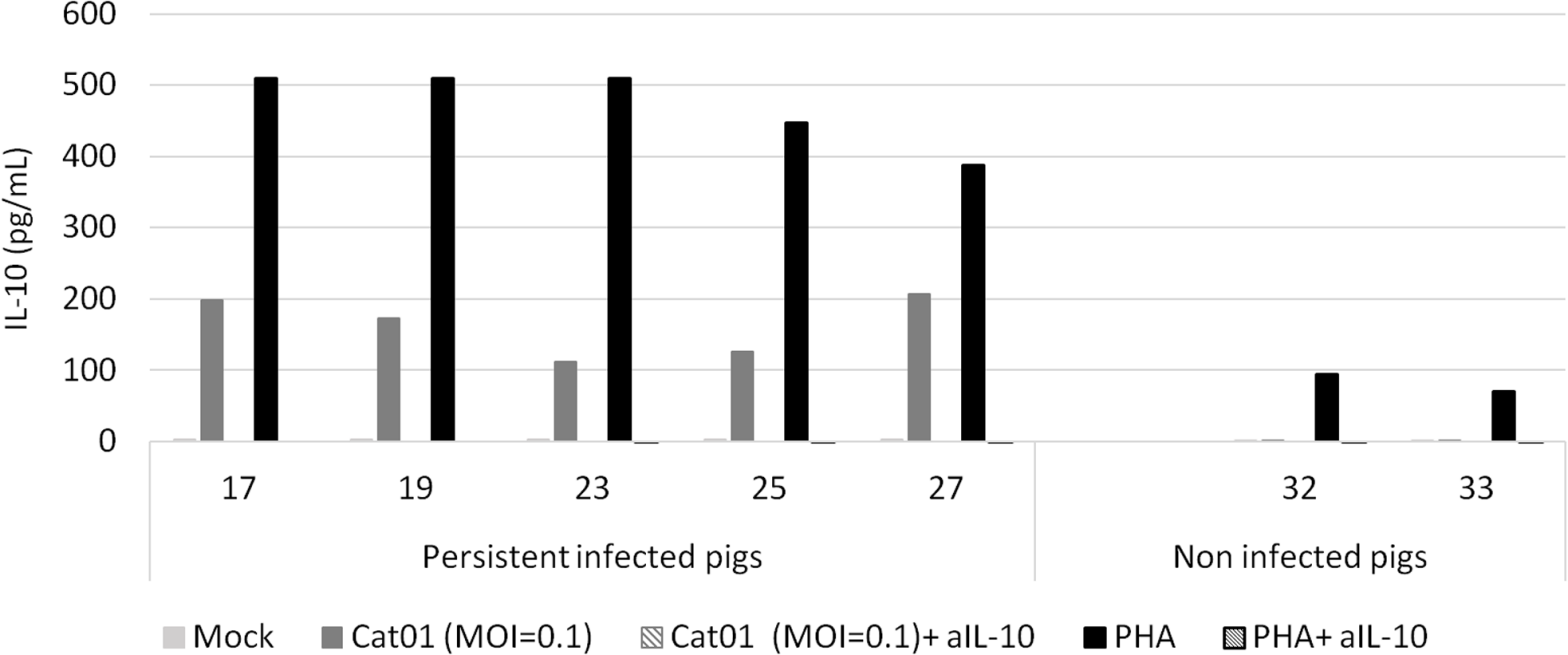
IL-10 levels detected by ELISA in the supernatants of PBMCs from CSFV-infected piglets developing the persistent form of CSF. PBMCs stimulated with Mock; CSFV Cat01 strain (MOI = 0.1) or PHA (1μg/ml) in the presence or absence of the anti-IL-10 neutralising Ab (aIL-10). PBMCs from pigs developing persistent CSF disease at 6 weeks post-infection (pigs 17, 19, 23, 25 and 27) and pigs 32 and 33 (non-infected pigs).

To determine the IL-10 levels in the sera of the persistent and immunocompetent piglets and of the dams, serum samples were analysed by ELISA at 7, 14, 21 and 42 dpi. IL-10 was not detected in any of the serum samples analysed (data not shown). In parallel, the IL-10 production of stimulated PBMCs from persistently infected piglets was analysed. PBMCs were collected from 6-week-old piglets that were either persistently infected with CSFV Cat01 (pigs 17, 19, 23, 25 and 27) or that were left uninfected as control (pigs 32 and 33). PBMCs from persistently infected piglets produced IL-10, while PBMCs from non-infected piglets did not (Fig 7). Importantly, PBMCs from persistently infected and from uninfected pigs responded to IL-10 production with PHA mitogen stimulation. None of the pigs had detectable levels of IL-10 in the supernatant of sham-stimulated cells (Fig 7).

### Defective CSFV-specific IFN-γ production by PBMCs from persistently infected piglets is not solely due to IL-10

**Fig 8:**
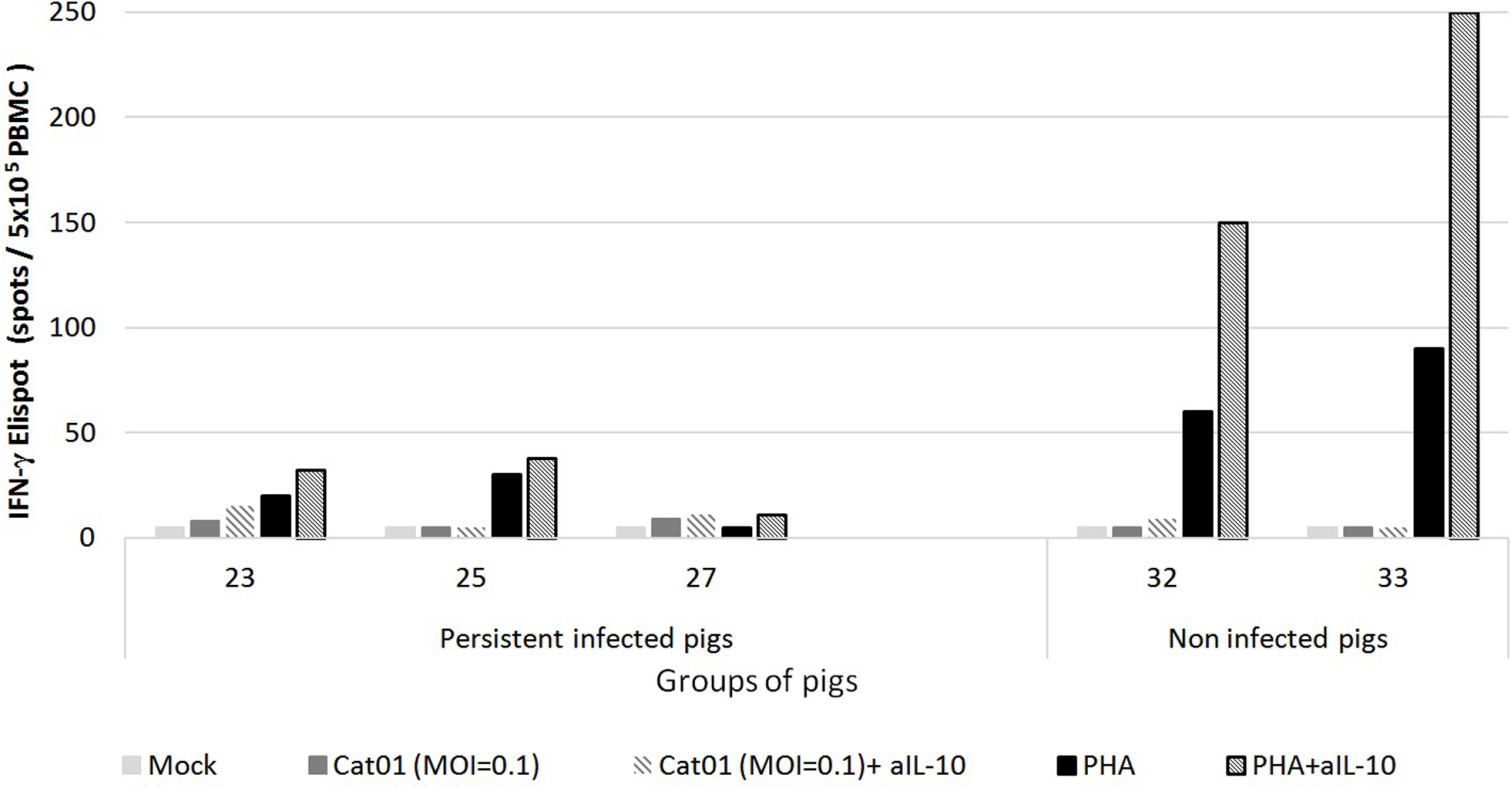
IFN-γ production in the presence or absence of the anti-IL-10 neutralising Ab in PBMCs from piglets persistently infected with CSFV. PBMCs from pigs developing persistent CSF at 6 weeks post-infection (pigs 23, 25 and 27) and pigs 32 and 33 (non-infected pigs) were stimulated with mock, with CSFV strain Cat01 (MOI = 0.1) or with PHA (10μg/ml) in the presence or absence of anti-IL-10 neutralising Ab (aIL-10).

Because IL-10 production could be stimulated with virus only in the PBMCs from the persistently infected piglets (Fig 7) and because there were no IFN-γ producing cells in the stimulated PBMCs from these animals (Fig 4C), the number of IFN-γ-producing cells upon CSFV stimulation was determined with or without the presence of the anti-IL-10 neutralising antibody (Ab). Additionally, the addition of anti-IL-10 Ab alone to PBMC cultures in the absence of antigen had no effect (data not shown). However, despite the anti-IL-10 Ab in the PBMC culture from persistently infected piglets, the response of the IFN-γ-producing cells was not restored (Fig 8). Nevertheless, IFN-γ production by PBMCs from non-infected piglets was significantly increased in the presence of anti-IL-10 neutralising Ab.

## Discussion

Despite intensive vaccination programs in some endemic countries, CSF has not been eradicated, due to failures in the responses to vaccination associated with poor handling of the vaccine, among other issues [4,5,7,16,17]. Moreover, the virus tends to evolve towards low virulence variants that circulate and persist in the pig population in association with common porcine infectious diseases [4,5,15]. CSFV persistence in offspring after trans-placental infection during mid-gestation has been well documented, contrary to postnatal infection [reviewed in [26]] and vaccination with CSFV live attenuated vaccine before the ingestion of colostrum, which conferred good protection against CSF in newborn pigs [17,45]. Here, we show that persistently infected piglets could be generated following infection with two different CSFV field isolates (of low and moderate virulence) on the day of their birth. These piglets remained healthy for several weeks, without any specific immunological response to CSFV and with high virus loads in the blood, organs and body secretions. In this context, persistently infected pigs might play an important role in virus dissemination. Some of these piglets developed fever peaks during the first 15 days post-infection and non-specific clinical signs and lesions, mostly associated with omphalophlebitis and secondary bacterial infections (confirmed at necropsy) which resulted in death or required euthanasia. On the other hand, severe thymus atrophy was the main gross pathological lesion (data not shown). Interestingly, all of the persistently infected pigs were CSFV RNA positive in the thymus. In contrast, a reduction in the number of PBMCs from these animals was also observed (data not shown). Thymus atrophy has been described in previous studies after CSFV congenital persistent infection with the Bergen strain [20,25]. However, thymus atrophy is not an exclusive finding of this form of the disease because it has also been described in the CSF acute form, wherein massive lymphoid depletion was also found due to lymphocyte apoptosis in atrophied thymuses [46]. In addition, previous studies have shown B-lymphocyte, helper T-cell and cytotoxic T-cell depletion during CSF acute disease [47]. Finally, thymus atrophy can also be caused by other viral infections, such as porcine circovirus-2 [48], porcine reproductive and respiratory syndrome virus [49,50] and influenza A virus [51], all of which are related to lymphoid depletion.

Owing to omphalophlebitis (a clinical sign not related to CSFV infection) developing in the pigs inoculated with the PR strain, the mortality was higher in this group. Nevertheless, the viral load in these piglets was consistently lower than the viral load detected after Cat01 strain infection. Considering as one virulence criterion the CSFV replication levels [12,32,52,53], our results might indicate lower virulence of the PR strain. Indeed, the three piglets inoculated with the PR virus became immunocompetent, clearing the virus from sera after three weeks post-infection, whereas none of the Cat01-infected piglets seroconverted. Furthermore, the sows from both groups were infected when in contact with their offspring. However, only the sow in the Cat01 group was CSFV-positive from rectal and nasal swabs during the last four weeks of the trial, being an asymptomatic carrier of the virus despite the neutralising antibody response.

Viral detection in some organs from the immunocompetent piglets was remarkable, and it might have been due to the low levels in the neutralising antibody titres detected from the fourth to the sixth weeks post-infection. Previous studies have shown that vaccine-challenged pigs that were not fully protected were CSFV positive in the tonsils after viral challenge, despite having some neutralising antibody response [7,54–56]. In contrast, the constant exposure of the immunocompetent piglets to the virus from the remainder of the infected litter likely influenced the neutralising antibodies’ consumption, thus avoiding the anamnestic effect of the humoral response [57].

Thus, the virulence of CSFV might be a critical factor in determining the outcome of early post-natal infection, considering the capacity of the Cat01 moderate virulence strain to induce persistent disease. Nevertheless, it is not known whether this outcome could also apply to trans-placental infections at mid-gestation. The proportion of persistently infected piglets could likely vary between reports with different viruses [13,20,21].

Compared to the adaptive immune response, the innate immune response to the virus, as measured by type I IFN-α in the serum, was not impaired in the persistently infected piglets. At seven days post-infection, when the viral loads were similar in all of the piglets, the IFN-α levels were comparable in the pigs that had seroconverted and in the persistently infected piglets (Fig 5). Similarly, serum IFN-α levels were measured in Cat01 virus-infected 10-week-old immunocompetent pigs in a separate study [32]. Surprisingly, the persistently infected piglets inoculated with the PR strain had overall significantly higher serum IFN-α levels than the Cat01-infected pigs, although their virus load was lower. This situation was different from acute CSFV infections, in which the serum IFN-α levels were consistently higher with higher virus titres in the circulation [58,59]. Additionally, it was recently demonstrated that E^rns^ impaired pDC-mediated IFN-α secretion in response to CSFV infection [60]. Different efficiencies of the E^rns^ of PR *versus* Cat01 in terms of pDC inhibition might account for the discrepancy observed here.

Four weeks after infection, the PBMCs from the persistently infected and seronegative piglets were unresponsive to both specific CSFV and non-specific PHA stimulation, in terms of IFN-γ producing cells. On the contrary, the three piglets that cleared the virus and seroconverted responded equally well to CSFV and PHA stimulation. PHA and Concanavalin A have been used previously to characterise the general functionality of the cellular immune response in the context of acute and persistent CSFV infections. After acute CSFV infections, the responses of PBMC to mitogens were also partly or completely impaired, indicating general transient immunosuppression [32,42,61]. In contrast, with trans-placental infections, immunotolerant pigs showed a normal lymphocyte response to PHA, indeed suggesting specific immunotolerance, rather than general immunosuppression [21,62,63].

IL-10 is a well-characterised immunosuppressive cytokine that inhibits a broad spectrum of immune responses, including the suppression of stimulatory cytokine production, T-cell proliferation, and B-cell responses [64–68]. A previous study provided the ability of a highly virulent CSFV strain to induce detectable levels of IL-10 in the serum of pigs developing the acute form at 7 days post-infection [69]. However, IL-10 was not detected in any of the serum samples analysed from the persistently infected piglets. This finding could suggest that different roles are played by this cytokine in the two CSF forms. Nevertheless, the CSFV- and PHA-stimulated PBMCs from persistently infected piglets produced high levels of IL-10. In this regard, hepatitis C virus (HCV), which is also a member of the *Flaviviridae* family, induces the production of IL-10 by cells of the innate immune system, principally by monocytes. This response has been associated with the suppression of the adaptive immune response in HCV persistently infected patients [66,70]. In this context, CSFV behaves in a similar manner to HCV, avoiding clearance by the immune system of the host. Moreover, the addition of neutralising IL-10 Ab did not restore the number of IFN-γ-producing cells in PBMCs from persistently infected piglets. Therefore, other mechanisms might also be involved in the general suppression of the T-cell response upon CSFV and mitogen activation. Previous studies have shown a drastic decrease in the T cell populations due to lymphocyte apoptosis during the acute form [59], and it could be another factor preventing the activation of the adaptive immune response in persistently infected animals, which also showed PBMC depletion.

Interestingly, the percentage of CD172a+/SLAII+ cells found in the BMHCs from persistent infected piglets was increased by 20% over the value found in naive pigs. Previous studies with the CSF acute form have shown that, after severe immunosuppression, SLAII+ cell populations decreased considerably [71]. Similarly, immature granulocytes, specifically 6D10+ cells [72], were the predominant cell population in these pigs, similar to the cellular profiles found after the CSF acute form [46,71]. These cells were infected with a high concentration of viral RNA, promoting virus spread in the persistently infected animals.

To the best of our knowledge, this is the first comprehensive study showing the ability of CSFV to generate viral persistence after early postnatal infection, which has not been described with other members of the *Pestivirus* genus either. In endemic areas where serological methods are used without virus detection in CSFV surveillance, postnatally persistently infected piglets would remain unnoticed. In addition to the epidemiological and economic significance of persistent CSFV infections, this model will be useful for understanding the mechanisms of viral persistence.
